# SPME-GC-MS and Multivariate Analysis of Sensory Properties of Cheese in a Sack Matured with Probiotic Starter Cultures

**DOI:** 10.17113/ftb.58.02.20.6439

**Published:** 2020-06

**Authors:** Deni Kostelac, Marija Vrdoljak, Ksenija Markov, Ivančica Delaš, Tjaša Jug, Jasenka Gajdoš Kljusurić, Željko Jakopović, Iva Čanak, Marko Jelić, Jadranka Frece

**Affiliations:** 1 Faculty of Food Technology and Biotechnology, Department of Biochemical Engineering, Laboratory for General Microbiology and Food Microbiology, Pierottijeva 6, 10000 Zagreb, Croatia; 2 Polytechnic ˝Marko Marulić˝, Petra Krešimira IV 30, 22 300 Knin, Croatia; 3 School of Medicine, University of Zagreb, Department of Chemistry and Biochemistry, Šalata 3, 10 000 Zagreb, Croatia; 4 Agricultural and Forestry Institute of Nova Gorica, Agrochemical Laboratory, Pri hrastu 18, 5000 Nova Gorica, Slovenia

**Keywords:** traditional cheese, cheese ripening, probiotic bacteria, starter culture, aromatic profiles

## Abstract

**Research background:**

Cheese in a sack is a traditional cheese produced in Croatia. Types of cheese with similar production technology are made in other countries but chemical and microbiological composition varies between regions. Traditionally, cheese in a sack is produced without the addition of starter cultures. Addition of beneficial probiotic cultures to numerous dairy products has documented advantages. Effects that the addition of probiotic bacteria to traditional cheese have on aroma compounds and sensory properties have not been fully investigated. The aim of this study is to determine the sensory properties and differences in the aromatic profiles between cheese samples ripened in a lambskin sack, produced traditionally without the addition of any starter culture, or with the addition of probiotic bacteria.

**Experimental approach:**

In this study, cheese in a sack was produced with the addition of probiotic cultures *Lactobacillus plantarum* B and *L. lactis* ssp. *lactis* S1. During ripening volatile aroma compounds were analysed with a solid-phase microextraction gas chromatography-mass spectrometry. Sensory properties were evaluated by trained tasters who are familiar with the traditional taste of the cheese from a sack. The results of aroma composition and taste scores were then compared using factorial and principal component analyses.

**Results and conclusions:**

Chromatography showed differences in the composition of aroma compounds and the sensory properties between the cheese produced with *Lactobacillus* starter cultures and the control cheese, traditionally produced without a starter culture. The addition of probiotic cultures *L. plantarum* B and *L. lactis* ssp. *lactis* S1 resulted in products with better sensory properties and chemical profile of volatile aromatic compounds.

**Novelty and scientific contribution:**

This study investigates the usage of naturally present probiotic cultures as starter cultures in cheese in a sack production. Their effects on aroma profiles and sensory characteristics have been compared for the first time using factorial and principal component analyses.

## INTRODUCTION

Cheese in a sack is highly regarded traditional cheese matured anaerobically in a lambskin sack (*1*), still produced in local communities in the south of Croatia (*2*). It is prepared from unpasteurised milk without the addition of starter cultures, so milk is acidified with its natural microflora. Even though the procedure has been adjusted to new technologies, the basic production parameters and cheese properties remain unchanged and unique.

Similar types of cheese in animal skin sack are still produced in Bosnia and Herzegovina (*3*) Montenegro (*1*), Turkey (*4*), Lebanon (*5*), and eastern Algeria (*6*). Chemical composition varies in different regions of production. Data from Croatian region indicate that the cheese composition is around 62% dry matter, 24% protein, 53% fat in dry matter and 4% salt with pH around 5 (*2*).

Not much data about their microbiological and organoleptic properties is available in the literature. What is known is that microbiological and sensory properties of dairy products, including aroma, can be enhanced by the addition of starter cultures such as lactic acid bacteria (*7*, *8*). The best way to preserve the specific properties of a traditional cheese is to use native starter cultures. However, while the addition of probiotic bacteria has been well described in common dairy production (*2*, *9*), only a few studies have looked into the subject with the traditional cheese in a sack. Probiotic bacteria are live microorganisms that, when administered in adequate amounts, confer a health benefit on the host (*10*). Used in dairy matrices, beneficial lactic acid bacteria have demonstrated positive health effects like the hypolipidaemic effects of symbiotic yoghurt containing *Lactobacillus acidophilus* in *in vivo* study (*11*) and notable *in vitro* antimicrobial and antidiabetic efficacy in diabetic rabbit study of fermented milk containing probiotic bacteria *Lactobacillus acidophilus* (*12*). High survival rates of beneficial bacteria during production and storage are of high importance in order to exhibit health benefits to the consumer. Furthermore, sensory quality of products with added bacterial cultures should remain high. Several studies have suggested that various food matrices support the added bacteria during production and storage and have a satisfactory sensory quality. Probiotic ice cream product retained high survival of bifidobacteria after freezing and during storage (*13*) and probiotic strains added to goat’s milk survived during 14-day storage and opened a possibility to affect sensory quality (*14*). Sensory characteristics and flavour of fermented products are of great importance to the consumer and have to be taken into consideration (*15*). Type of milk and cheese production, together with added microorganisms play an important role in sensory quality and stability during storage. It was demonstrated that probiotic cow’s milk yoghurts scored better sensory quality than goat’s milk yoghurts with added probiotic culture after 45 storage days (*16*). Cheese types with probiotic cultures have demonstrated improved quality and health benefits. For example, Tulum cheese produced with probiotic cultures achieved higher flavour scores and better texture (*17*), and Prato cheese proved to be great matrix for probiotic delivery and demonstrated health benefits such as reduction of oxidative stress (*18*) and attenuation of renal calculi (*19*) in animal models. Furthermore, the addition of probiotic bacteria during production of processed cheese ’requeijão cremoso’ prevented final product recontamination (*20*).

The aim of this study is to investigate how the addition of pure culture of strains *Lactobacillus plantarum* B and *Lactococcus lactis* ssp. *lactis* S1, native to the cheese in a sack, affects organoleptic properties compared to the control cheese made traditionally, without these probiotic cultures. We compared the properties of the control cheese to those of the cheese started with either one or both probiotic strains, at different stages of ripening (15, 30 and 45 days). To make the comparison as informative as possible and classify the cheese samples based on their aromatic components and sensory properties, we introduced for the first time two multivariate analyses: factorial and principal component.

## MATERIALS AND METHODS

### Cheese making procedure

To make the cheese, we used 80 L of a mix of the evening and morning unpasteurised milk of the Dalmatian Pramenka sheep breed obtained on local farms near the city of Knin, Croatia. The cheese was produced following the technology for semi-hard cheese production. The control cheese was made without the starter probiotic culture (NC), one was made with *Lactobacillus plantarum* B (LP), one with *Lactococcus lactis* ssp. *lactis* S1 (LL), and one with a mix of both cultures in the 1:1 ratio (MC). All the applied strains were previously isolated from the traditionally prepared cheese (*9*) and characterised as probiotic cultures (*2*).

The control cheese was started with commercial rennet (Maxiren^®^ 1800; Royal DSM, Heerlen, The Netherlands) according to the manufacturer's instructions at 32 to 33 °C. Cheese curd was then cut into 2 cm×2 cm cubes with a clean knife. The curd grains were stirred and heated up to between 38 and 39 °C. After drying, the size of the curd grains corresponded to the size of a pea. Clumps of raw cheese were formed manually in the whey, and put into plastic sieves. After filtering, the cheese was cut into pieces, salted with sea salt, stuffed in a lambskin sack, also obtained at the farm near the city of Knin, and left to ripen at 14 to 16 °C and relative humidity of 65 to 80% for 45 days.

For the cheese started with probiotic cultures, the milk was warmed to 32 to 33 °C, the cultures were added as wet biomass of 1.5 g, and left to rest for half an hour. Then we added commercial rennet, and the rest of the procedure was the same as in the production of the control cheese. The lambskin sacks were prepared as described in detail by Frece *et al*. (*9*).

### Analysis of volatile aroma compounds

#### Sample preparation

Cheese was sampled throughout the ripening time on the 15th, 30th and 45th day. After each sampling, the skin sack was tied up tightly, and stored again in a ripening chamber. Cheese samples were stored at -80 °C until analysis. The surface portion of the cheese (approx. 0.5 cm) was discarded; 5 g of cheese sample were ground in a blender and transferred into a 15-mL headspace vial. Then, 5 μL of 1-octanol (Merck, Darmstadt, Germany) as an internal standard were added, and allowed to equilibrate at 40 °C for 15 min. All samples were taken in triplicate.

#### Solid-phase extraction

Samples were prepared for gas chromatography by solid-phase microextraction (SPME) using 50/30 divinylbenzene/carboxen/polydimethylsiloxane (DVB/CAR) on PDMS fibre (Supelco, Bellefonte, PA, USA) (*21*, *22*).

#### Gas chromatography-mass spectrometry

Extracted volatile compounds were analysed by a modified method of gas chromatography-mass spectrometry (*22*, *23*). The sample was injected into a gas chromatograph with a mass detector (GC-MS; Agilent 6890 Series GC system with Agilent 5973 mass selective detector; Agilent, Santa Clara, CA, USA). The injector temperature in splitless mode was 270 °C, and the desorption time was 10 min. Volatile aromatic compounds were separated on Rtx-20 column (60 m×0.25 mm i.d., film thickness 1 μm; Restek, Bellefonte, PA, USA) using the following 30-minute temperature programme: 50 °C for 2 min, ramp to 150 °C at the rate of 10 °C per min, 150 °C for 3 min, ramp to 250 °C at 10 °C per min, and finally 250 °C for 5 min.

The GC-MS working conditions were as follows: electron ionisation 70 eV, quad temperature 150 °C and ion source 230 °C. Volatile aroma components were identified using AMDIS program v. 3.2 (*24*), based on their retention time (*t*_R_) and mass spectra (MS) provided by the National Institute of Standards and Technology (NIST) software (*25*). The peak area for quantification was measured in total ion chromatogram. The results are expressed as the percentages of each identified volatile compound in relation to the total quantity of identified volatile compounds.

### Sensory assessment

The sensory properties of the cheese matured in a sack after 15, 30 and 45 days were evaluated by a group of trained local consumers (*N*=10, 5 male, 5 female, aged 25-40), familiar with this type of cheese, and trained for sensory evaluation at the Polytechnic Marko Marulić in Knin, Croatia. The appearance (0.5-2), colour (0.5-1), body (0.5-3), texture (0.5-2), aroma (0.5-2) and taste (0.5-10) of cheese samples were evaluated according to the scoring criteria established at the Faculty of Agriculture, University of Zagreb (*3*). The highest achievable total score was 20.

### Statistical analysis

For all parameters, we used the average values of triplicate measurements. The data matrix was constructed from the columns showing aromatic breakdown (66 compounds presenting the input variables) of the four sack cheese varieties at the three sampling days (15, 30 and 45 days). Thereby, the input data matrix had in total 66 rows and 80 columns. For such data set it is common to analyse interactions in the observed dataset, that is assay all independent variables simultaneously. Autoscaling, as the most commonly used pre-processing in chemometrics, was applied to produce variables with zero means and unit standard deviation (*26*). To establish which compounds differed significantly between the varieties, we used the multivariate analysis of variance (MANOVA), which included factorial analysis (FA), and principal component analysis (PCA), where PCA is a one of the forms of FA. In accordance with the study with similar methodology, FA was used to reduce the initial set of 66 components to 17 principal components with significant differences, based on a factor score greater than 0.7 because values under 0.5 will not be useful (*27*, *28*). To obtain as much information as possible from the extracted principal components, we used varimax rotation. Linear discriminant analysis (LDA) was used to group aroma components according to the cheese type and ripening days. All statistical analyses were performed using TIBCO Statistica, v. 13.3.0 (*29*).

## RESULTS AND DISCUSSION

Sensory differences in aroma, texture, body and taste were analysed during 45 days of ripening between the cheese samples with added cultures and the traditionally produced cheese (Table 1).

**Table 1:** Sensory scores of cheese in a sack during 45 days of ripening

Property	*t*(ripening)/ day		NC	LL	LP	MC
Appearance		15	1.95^a^	2^a^	2^a^	1.93^a^
Colour			1^a^	1^a^	1^a^	1^a^
Texture			1.38^a^	1.83^b^	1.98^b^	1.93^b^
Body			2.38^a^	2.8^b^	2.98^b^	2.93^b^
Odour			1.88^a^	1.93^a^	1.98^b^	1.85^a^
Taste			6^a^	8.2^b^	9.43^b^	9.05^b^
Total			14.58^a^	17.75^c^	19.35^b^	18.09^c^
Appearance		30	1.65^a^	1.8^a^	2^b^	2^b^
Colour			0.9^a^	0.78^b^	1^a^	1^a^
Texture			1.2^a^	1.85^b^	1.98^b^	1.93^b^
Body			2.56^a^	2.78^a^	2.98^b^	2.95^b^
Odour			1.15^a^	1.78^b^	2^b^	1.95^b^
Taste			1.56^a^	9.38^b^	9.73^c^	8.93^b^
Total			12.45^a^	18.35^c^	16.97^b^	18.75^c^
Appearance		45	1.68^a^	1.85^a^	2^b^	2^b^
Colour			0.8^a^	0.85^a^	0.93^a^	1^b^
Texture			1.13^a^	1.95^b^	2^b^	1.98^b^
Body			2.38^a^	2.83^a^	3^a^	2.98^a^
Odour			1.2^a^	1.85^b^	1.83^b^	1.93^b^
Taste			4.95^a^	9.38^b^	9.68^b^	9.5^b^
Total			12.14^a^	18.71^c^	19.44^b^	19.39^b^

*Different letter in a row indicates significant difference (p<0.05). NC=no starter culture, LL=*Lactobacillus lactis* ssp. *lactis* S1, LP=*Lactobacillus plantarum* B and MC=mixed starter culture of *L*. *lactis* and *L*. *plantarum* (1:1 ratio)

Our findings suggest that cheese in a sack is a suitable matrix for the use of probiotic cultures, and that the addition of native cultures improves its sensory properties, which is consistent with the report by Soeryapranata *et al*. (*7*). They found that the cheese started with *Lactobacillus helveticus* WSU 19 was less bitter than the cheese without starter culture. Enzymes originating from the starter cultures (proteinases and peptidases) play a major role in the formation of small peptides and amino acids that serve as precursors for the compounds responsible for cheese flavour (*30*). In addition, Tudor Kalit *et al*. (*31*) have shown that ripening for a longer time (60 days) results in less acceptable products due to intensive proteolysis and lipolysis.

Table 2 shows the content of volatile aroma compounds of each of the four cheese samples.

**Table 2:** Volatile aroma compounds (*φ*/%) identified in the four cheese samples ripened in a sack for 15, 30 and 45 days

Volatile compound	No culture			*Lactococcus lactis* ssp. *lactis* S1			*Lactobacillus plantarum* B			Mixed culture (1:1 ratio)		
15	30	45	15	30	45	15	30	45	15	30	45
Acid												
Butyric	1.65	3.2	6.44	1.69	2.79	4.86	2.35	2.43	1.69	3.25	3.3	7.43
Acetic	1.1	5.85	11.5	3.33	0.6	6.93	7.65	9.29	9.98	7.4	9.73	5.29
3-Methylbutanoic	1.02	0.62	0.72	2.14	0.6	1.65	0.54	1.3	0.62	0.89	1.02	2.25
2-Methylbutanoic	0.37	0.28	0.25	0.75	0.17	0.98	0	0.52	0.21	0.29	0.35	1.01
Caproic	2.44	2.88	4.64	4.07	3.52	6.36	2.14	2.55	2.85	4.14	4.86	9.65
Caprylic	1.46	1.02	1.24	1.67	1.67	2.41	1.16	1.01	0.98	0.95	1.67	2.05
Alcohol												
Ethanol	15.52	4.94	8.26	9.35	6.08	8.85	14.81	17.15	12.51	16.47	15.2	4.48
2-Methyl-1-propanol	0.62	3.19	3.44	0.27	0	0	0.24	0.27	0.26	0	0.35	0.29
1-Butoxy-2-propanol	0	0	0.37	0.6	0.09	0	0.2	0.28	0.41	0.12	0.16	0.3
3-Methyl-1-butanol	4.44	10.34	1.84	3.83	3.8	3.84	4.43	4.75	3.69	1.92	4.15	3.23
2-Methyl-1-butanol	0.91	2.97	0.35	0.6	0.69	0.91	0	0.36	0	0	0.44	0
1-Hexanol	0.69	0	0	0	0.19	0.19	0.44	0.51	0	0	0.35	0
2-Butanol	0	0	0	0	0	1.27	0	0	0	0	0	0
2-Pentanol	0	0	0	0	0.34	0	0	0	0	0	0	0
Aldehyde												
Pentanal	1.31	0.48	0	0.28	0.19	0	0	0.37	0	0	0	0
Hexanal	5.2	2.66	1.84	3.16	1.76	0	0	3.25	2.92	1.97	2.72	0
Heptanal	3.7	1.89	0.37	0.75	0.63	0.24	0.53	0.85	0.71	0.41	0.45	0.2
Nonanal	0.45	0.31	0.23	0.23	0.72	0	0.27	0.24	0.36	0.22	0.18	0.14
Octanal	0.46	0.23	0	0	0.35	0	0	0.32	0.41	0.23	0.28	0
Ester												
Ethyl acetate	2.48	5.85	12.0	2.71	6.08	14.01	1.6	1.97	1.92	1.52	1.61	1.24
Ethyl butanoate	1.72	1.34	1.56	3	2.37	3.73	1.61	2.9	2.13	2.99	3.56	4.38
2-Methylpropyl butanoate	0.12	0	0	0.19	0.1	0.39	0	0.29	0.11	0.19	0.22	0.3
3-Methylbutyl ethanoate	0.54	11.6	0.64	0.32	0.18	2.06	0.37	0.62	0.49	0.44	0.32	0.71
1-Butanol-3-methyl acetate	0.24	2.64	0	0.42	0.72	0.6	0	0.67	0.22	0.46	0.51	0.68
Ethyl caproate	3.74	2.35	1.15	4.06	4	6.47	3.18	3.92	2.58	6.55	5.7	5.41
Ethyl caprylate	0.66	0.58	0.81	1.4	0.84	2.06	1.89	0.87	0.98	1.18	1.19	1.72
Ketone												
2-Butanone	0.11	0	0	0.15	7.01	0.84	0.29	0	0	0	0	0
2,3-Pentanedione	0.36	0	0.08	0.13	0	0	0.13	0.2	0	0	0	0
3-Hydroxy-2-butanone	1.69	2.31	2.47	0.92	0	0.38	5.75	2.27	6.02	3.02	1.11	4.83
6-Methyl-5-hepten-2-one	0	0	0	0.15	0.14	0	0	0	0.15	0	0	0
2,3-Butanedione	0	0	0	0	0	0	1.38	0.64	1.02	0.47	0.31	0.62
2-Nonanone	0	0	0	0	0	0	0.22	0.28	0.25	0	0	0.13
2-Heptanone	0	0	0	0	0	0	0	0.88	0	0	0	0
2-Pentanone	0	0	0	0	0	0	0	0.17	0	0	0	0.12
Terpene												
d-Limonene	6.25	0.83	1.16	1.89	2.83	0.37	1.88	1.59	2.48	1.38	1.27	1.43
Menthol	0	0	0	0	0.2	0	0	0	0	0	0	0
Carveol	0	0	0	0	0.41	0	0	0	0	0	0	0
Geraniol	0	0	0	0	1.61	0	0	0	0	0	0	0
α-Myrcene	0	0	0	0	0	0	0.58	0	0	0	0	0
α-Phellandrene	0.32	0	0	0	0	0	1.02	0	0.5	0.83	0	0
α-Pinene	0	0	1.65	0	0	0	0	0	0	0.74	0.74	0.66
*p*-Cimene	0	0	0.72	0.36	0.46	0.16	0.4	0.39	0.55	0	0	0.32
β-Cimene	0.29	0	1.94	0	0	0	0	0.7	0.69	0	0	0
Other												
Heptane	0.16	0	0	0.2	0.21	0	0.13	0.3	0	0	0.28	0
Decane	0	0	0	0.24	0.22	0	0.24	0	0.16	0	0	0
3,7-Dimethyl-1,6-octadiene	1	0.67	0.46	1.85	0.91	1.07	0.6	1.05	0.79	0.71	0.79	0.53
3,7-Dimethyl-2-octene	1.11	0.9	0	1.68	0	1.15	0.83	1.18	0.71	0.21	1.24	0.48
Nonane	0.07	0	0	0	0.17	0	0	0	0	0	0	0
2,2,4,6,6-Pentamethyl heptane	0.59	1.1	0	0	0.33	0.33	4.52	0.23	0.82	2.76	0.3	0.58
Undecane	0	0	0	0	0.26	0	0	0.19	0	0	0.17	0
2,2,4-Trimethylpentane	0	0	0	0	0.18	0	0	0	0	0	0	0
Tetradecane	0	0	0	0	0.55	0	0.16	0	0	0	0.15	0
3-Octene	0	0	0	0	1.7	1.82	0	3.08	0	0	2.71	0
5-Ethyl-2,2,3-trimethyl heptane	0	0	0	0	0	0	0.31	0	0	0	0	0
2,3,6,7-Tetramethyloctane	0	0	0	0	0	0	0.77	0	0	0	0	0
2,7,10-Trimethyldodecane	0	0	0	0	0	0	0.28	0	0	0	0	0
2,4,6-Trimethylheptane	0	0	0	0	0	0	0	0	0.17	0	0	0
2,6,10-Trimethyltetradecane	0	0	0	0	0	0	0	0	0.37	0	0	0
Toluene	0.65	0	0.51	1.27	0.63	0.6	0.67	0.85	0.97	0.74	0.75	0.13
2,5-bis(3-methylsiloxy) benzaldehyde	0.86	0	0.71	0.77	0	0.26	0.69	0	1.04	0.68	0	0.51
*p*-Xylene	1.03	0.95	0.26	0.64	1.19	0.24	0.3	0	0.5	0.46	0.35	0.34
*o*-Xylene	0	0	0	0	0.31	0	0	0.38	0	0	0	0
Styrene	0.58	0.48	0	0	0	0	0	0	0	0.32	0	0
Methoxy-phenyl-oxime	3.12	1.15	5.83	5.84	1.74	2.02	2.25	1.53	2.81	4.42	2.23	2.35
Carbon dioxide	0.38	0.4	0.36	0.38	0.39	0.24	0.34	0.37	0.36	0.26	0.37	0.36

**Fig. 1:**
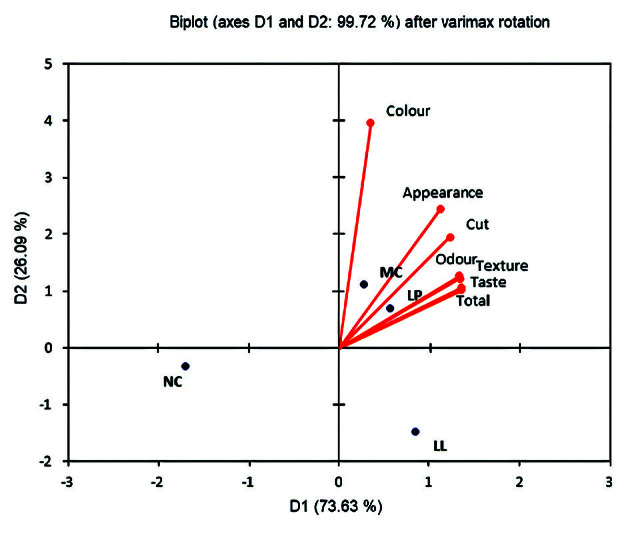
Biplot after varimax rotation of principal component analysis, based on the sensory properties of the four cheese samples: NC=no starter culture, LL=cheese produced with *Lactococcus lactis* ssp. *lactis* S1, LP=cheese produced with *Lactobacillus plantarum* B and MC= mixed starter culture of *L*. *lactis* and *L*. *plantarum* (1:1 ratio)

Fig. 1 shows the relationship between aroma scores and other sensory properties of the four cheese samples. The biplot shows a dominance of the cheese samples started with *Lactobacillus plantarum* (LP) and mixed cultures, since these samples received the highest scores for all sensory parameters (the first quadrant of the biplot). On the contrary, the cheese without starter culture (NC) received the worst sensory scores (3rd quadrant). Cheese produced with *L. lactis* ssp. *lactis* (LL) starter culture had lower scores (2nd quadrant) than the cheese with LP, but still fared better than the control cheese (judging by its position in the 3rd quadrant).

After the grouping was confirmed, we looked for the aroma components that caused such distribution. As some aroma components were not detected or were equal to zero, instead of MANOVA, we applied factor analysis to reduce the data and to identify those aroma compounds that significantly contributed to the sensory properties (*32*, *33*). From the set of 66 aroma components, we obtained 17 that significantly affected the sensory properties of the cheese samples (Table 3).

**Table 3:** Aroma components that significantly contributed to the sensory properties of the analysed cheese samples (factor score>0.7).

Component	NC (control)	LL	LP	MC
Lactic acid	+	+		+
Acetic acid	+	+	+	+
Caproic acid	+	+	+	+
Ethanol	+	+	+	+
2-Methyl-1-propanol	+			
3-Methyl-1-butanol	+	+	+	+
Hexanal	+			
Ethyl acetate	+	+	+	+
Ethyl butanoate		+		+
3-Methylbutyl ethanoate	+			
Ethyl caproate		+	+	+
2-Butanone		+		
3-Hydroxy-2-butanone			+	
2-Heptanone				
β-Cymene				
2,2,4,6,6-Pentamethyl-heptane				
Methoxy-phenyl-oxime	+	+		+

NC=no starter culture, LL=*Lactobacillus lactis* ssp. *lactis* S1, LP=*Lactobacillus plantarum* B and MC=mixed starter culture of *L*. *lactis* and *L*. *plantarum* (1:1 ratio)

These results, however, take all the samples as one data set, yet one of the aims was to profile the aroma compounds based on the added starter culture. This led to further reduction to eight significant aroma compounds for the cheese started with LP (lactic acid, caproic acid, ethanol, 2-methyl-1-butanol, ethyl acetate, ethyl caproate, 2-butanone and methoxy-phenyl-oxime), nine for the cheese produced with mixed starter cultures (lactic acid, acetic acid, caproic acid, ethanol, 2-methyl-1-butanol, ethyl acetate, ethyl butanoate, ethyl caproate, methoxy-phenyl-oxime), and ten for the cheese started with LL (lactic acid, acetic acid, caproic acid, ethanol, 2-methyl-1-butanol, ethyl acetate, ethyl butanoate, ethyl caproate, 2-butanone, methoxy-phenyl-oxime) (Table 3).

At higher concentrations, acetic acid is known to render cheese sour and pungent, which is what happened to the control (NC) cheese, whose acetic acid volume fraction rose from the initial 1.10 to 11.58% by the end of ripening. This is most likely the reason why it received the lowest score for aroma of all the cheese samples, and argues in favour of using starter cultures like ours, which seems to prevent acetic acid concentrations from rising too high. Hayaloglu *et al*. (*34*) reported similar observations and showed that the use of starter cultures significantly changed the ratio of acetic acid during the ripening of goat’s cheese.

The most common alcohol in our cheese samples was ethanol, identified in all the samples on all days of ripening, but at varying levels (Table 2). Ethanol is mainly formed by the fermentation of lactose, as well as by the alanine catabolism, and plays an important role in the formation of esters (*34*). It is the most common alcohol in the Tulum cheese, which also ripens in an animal skin (*35*), the feta cheese (*36*) and Halloumi (*37*).

Another alcohol common to all our cheese samples on day 45 of ripening was 3-methyl-1-butanol (or isoamyl alcohol), which is a product of the leucine metabolism (*38*). It has been identified by Serhan *et al*. (*39*) in the Lebanese goat Darfiyeh cheese, which also ripens in an animal skin. In a reaction with acetic acid, it produces isoamyl acetate, which has a pleasant fruity aroma of a pear or a banana. As its content on day 45 was significantly higher in treated than in control (NC) cheese, this could explain some of their higher taste scores.

Caproic acid is also considered to be responsible for the pungent taste (*40*). It has been identified in all samples on all tested days, which is not surprising, since it has been identified in many different types of cheese, especially those ripened in an animal skin (*35*, *41*). Its content increased with time in all but the LL cheese, where it dropped on day 30. However, the lowest volume fractions were observed in the LP cheese, which might have contributed to its highest sensory score on day 45.

All the cheese samples also contained ethyl caproate, albeit in varying fractions over time. Ethyl caproate is usually associated with a powerful fruity aroma of apple, grape, melon and pineapple. On day 45, we observed a significantly higher volume fraction of ethyl caproate in the LL cheese than in the control cheese. Ethyl esters were the predominant esters in the analysed samples due to the high volume fraction of ethanol arising from lactose fermentation or amino acid catabolism. The increase in the volume fraction of these esters could be responsible for the decrease in the volume fraction of the corresponding acids (*42*).

Ketones have a unique flavour and aroma, conferring mushroom-like and ’sweet’ notes to cheese. In our study, ketones most probably served as precursors for other compounds, because their volume fractions varied throughout ripening, which is consistent with the research of other authors (*43*, *44*).

Variations in the volume fraction of 3-hydroxy-2-butanone (acetoin) were observed in all samples throughout ripening, with a trend to increase in the control cheese. Acetoin is associated with a mushroom-like, sweet aroma of cheese. Its volume fraction was impermanent in our study, which can be explained by the fatty acid oxidation (*45*) or by the fact that acetoin serves also as a precursor for some secondary alcohols (*41*).

**Fig. 2:**
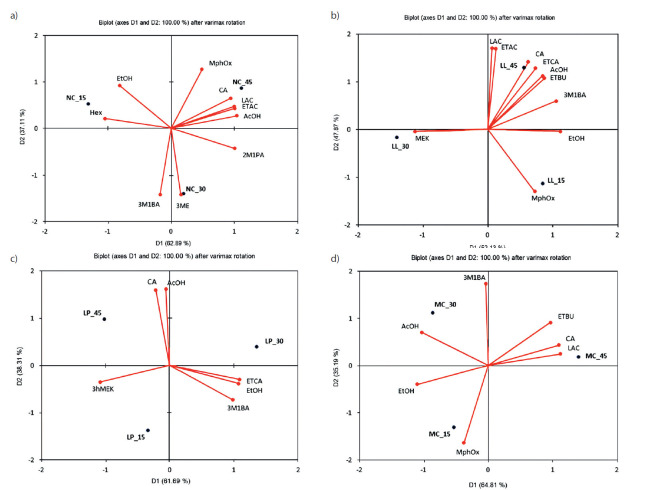
Biplots of the aroma compounds in the cheese produced: a) without added culture, and with added probiotic cultures: b) LL=*Lactobacillus lactis* ssp. *lactis* S1, c) LP=*Lactobacillus plantarum* B, and d) MC=mixed starter culture of *L*. *lactis* and *L*. *plantarum* (1:1 ratio) after 15, 30 and 45 days of ripening. LAC=lactic acid, AcOH=acetic acid, CA=caproic acid, EtOH=ethanol, 2M1PA=2-methyl-1-propanol, 3M1BA=3-methyl-1-butanol, Hex=hexanal, AC=ethyl acetate, ETBU=ethyl butanoate, 3ME=3-methylbutyl ethanoate, ETCA=ethyl caproate, MEK=2-butanone, 3ohMEK=3-hydroxy-2-butanone, 2HPONE=2-heptanone, bCyN=β-cymene, PMHe=2,2,4,6,6-pentamethyl-heptane, MPhOx=methoxy-phenyl-oxime

Fig. 2 shows the prevalent distribution of 17 aroma components for each of our cheese samples.

Components in the same quadrant for each observed cheese sample are prevalent in that cheese (see Table 2 for details).

Seven aroma components: lactic acid (LAC), ethyl acetate (ETAC), caproic acid (CA), ethyl caproate (ETCA), acetic acid (AcOH), ethyl butanoate (ETBU) and 3-methyl-1-butanol (3M1BA) in the first quadrant with the LP cheese (Fig. 2b) suggest that they are the most responsible for its best sensory score (19.44).

**Fig. 3:**
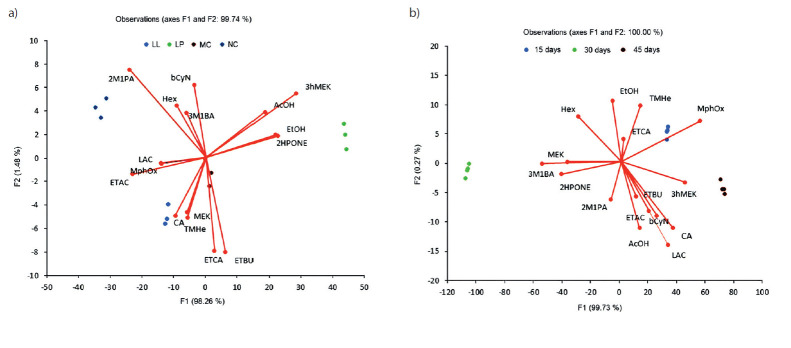
Distribution of prevalent aroma components by: a) the cheese and b) the days of ripening. Aroma components: LAC=lactic acid, AcOH=acetic acid, CA=caproic acid, EtOH=ethanol, 2M1PA=2-methyl-1-propanol, 3M1BA=3-methyl-1-butanol, Hex=hexanal, AC=ethyl acetate, ETBU=ethyl butanoate, 3ME=3-methylbutyl ethanoate, ETCA=ethyl caproate, MEK=2-butanone, 3ohMEK=3-hydroxy-2-butanone, 2HPONE=2-heptanone, bCyN=β-cymene, PMHe=2,2,4,6,6-pentamethyl-heptane, MPhOx=methoxy-phenyl-oxime; NC=no starter culture, LL=*Lactobacillus lactis* ssp. *lactis* S1, LP=*Lactobacillus plantarum* B and MC=mixed starter culture of *L. lactis* and *L. plantarum* (1:1 ratio)

Having identified the important aroma compounds and associating them with the starter cultures (LP, LL and MC), we further tested whether the identified 17 aroma components could be used to predict which culture would yield the best results, and give sensory assessment based on the days of ripening. With this idea in mind, we designed linear discriminant analysis (LDA) with principal components (Fig. 3).

Efficient application of the chemometric analysis for investigating the correlations between chemical composition and aroma (*46*), sensory, or different observed properties (*27*), is now improved with this study. Results presented in Fig. 3 show the efficient cheese classification based on the preparation process (Fig. 3a), *i.e.* without (NC) or with added starter probiotic culture (LP, LL and MC), as well as on the observation on different ripening days (15, 30 or 45; Fig. 3b). These results, which arise from the applied multivariate tools, related a sample’s aroma attributes with two important factors in cheese production: probiotic cultures and the time of ripening. Despite the cheese differences, data processing showed the observed aroma information as useful for sample discrimination. Further sensory tests as projective method (*47*) and innovative test based in consumer perception (*48*, *49*) should be performed.

## CONCLUSIONS

This study investigated the addition of probiotic cultures *Lactobacillus plantarum* and *L. lactis* ssp. *lactis* S1 to the cheese in a sack during production and their effect on organoleptic properties. We compared the properties of the control cheese to those of the cheese started with either one or both probiotic strains, at different stages of ripening. Two multivariate analyses: factorial and principal component analyses were conducted in the investigation of correlations between cheese composition, aroma and sensory properties. The addition of probiotic cultures *L. plantarum* and *L. lactis* ssp. *lactis* S1 improved the aromatic profile of the cheese in a sack, since they showed better sensory properties than the control cheese (without added culture). Additionally, they did not change the authentic taste of the traditional cheese.
